# Secreted protein acidic and rich in cysteine (SPARC) knockout mice have greater outflow facility

**DOI:** 10.1371/journal.pone.0241294

**Published:** 2020-11-04

**Authors:** Ling Yu, Yuxi Zheng, Brian J. Liu, Min Hyung Kang, J. Cameron Millar, Douglas J. Rhee

**Affiliations:** 1 Department of Ophthalmology & Visual Sciences, University Hospitals Eye Institute, Case Western Reserve University School of Medicine, Cleveland, Ohio, United States of America; 2 Department of Ophthalmology, the Affiliated Hospital of Southwest Medical University, Luzhou, Sichuan Province, China; 3 Department of Pharmacology & Neuroscience, North Texas Eye Research Institute (NTERI), University of North Texas Health Science Center, Fort Worth, Texas, United States of America; Bascom Palmer Eye Institute, UNITED STATES

## Abstract

**Purpose:**

Secreted protein acidic and rich in cysteine (SPARC) is a matricellular protein that regulates intraocular pressure (IOP) by altering extracellular matrix (ECM) homeostasis within the trabecular meshwork (TM). We hypothesized that the lower IOP previously observed in *SPARC -/-* mice is due to a greater outflow facility.

**Methods:**

Mouse outflow facility (C_live_) was determined by multiple flow rate infusion, and episcleral venous pressure (P_e_) was estimated by manometry. The animals were then euthanized, eliminating aqueous formation rate (F_in_) and P_e_. The C value was determined again (C_dead_) while F_in_ was reduced to zero. Additional mice were euthanized for immunohistochemistry to analyze ECM components of the TM.

**Results:**

The C_live_ and C_dead_ of *SPARC -/-* mice were 0.014 ± 0.002 μL/min/mmHg and 0.015 ± 0.002 μL/min/mmHg, respectively (*p =* 0.376, N/S). Compared to the C_live_ = 0.010 ± 0.002 μL/min/mmHg and C_dead_ = 0.011 ± 0.002 μL/min/mmHg in the WT mice (*p =* 0.548, N/S), the C_live_ and C_dead_ values for the *SPARC -/-* mice were higher. P_e_ values were estimated to be 8.0 ± 0.2 mmHg and 8.3 ± 0.7 mmHg in *SPARC -/-* and WT mice, respectively (*p =* 0.304, N/S). Uveoscleral outflow (F_u_) was 0.019 ± 0.007 μL/min and 0.022 ± 0.006 μL/min for *SPARC -/-* and WT mice, respectively (*p =* 0.561, N/S). F_in_ was 0.114 ± 0.002 μL/min and 0.120 ± 0.016 μL/min for *SPARC -/-* and WT mice (*p =* 0.591, N/S). Immunohistochemistry demonstrated decreases of collagen types IV and VI, fibronectin, laminin, PAI-1, and tenascin-C within the TM of *SPARC -/-* mice (*p* < 0.05).

**Conclusions:**

The lower IOP of *SPARC -/-* mice is due to greater aqueous humor outflow facility through the conventional pathway. Corresponding changes in several matricellular proteins and ECM structural components were noted in the TM of *SPARC -/-* mice.

## Introduction

Primary open-angle glaucoma (POAG) is one of the leading causes of blindness throughout the world, affecting approximately 70 million people [1–3]. The major causative risk factor for POAG is an elevated intraocular pressure (IOP) [4–6]. Elevated IOP is a result of increased resistance to aqueous humor outflow through the trabecular meshwork (TM) [7–11]. The equilibrium between extracellular matrix (ECM) deposition, modification, and turnover is essential for regulating resistance to outflow in the TM [12–14]. The molecular mechanisms controlling ECM homeostasis are not fully elucidated.

Matricellular proteins are nonstructural, secreted glycoproteins that facilitate cellular control over their surrounding ECM [15]. The matricellular protein Secreted Protein Acidic and Rich in Cysteine (SPARC) is one of the most highly transcribed genes in TM tissue and in TM cells undergoing physiological stress, indicating the important role of SPARC in normal physiology [16–18]. SPARC is present in many tissues throughout the body and has a prominent role in ECM homeostasis, especially fibrosis [19,20]. SPARC overexpression in perfused cadaveric human anterior segments increased IOP with a corresponding increase of fibronectin and collagen IV in the juxtacanalicular (JCT) TM. This increase of fibronectin and collagen IV was also observed in cultured human TM endothelial cells [21].

Transforming growth factor-β2 (TGF-β2) has been implicated in the pathogenesis of POAG [22–31]. In TM cells, SPARC is up-regulated by TGF-β2 via the smad 2/3, JNK, and p38 pathways [32,33]. The transgenic deletion of SPARC in mice prevents the ocular hypertensive effects of TGF-β2 [34]. We have previously shown that *SPARC -/-* mice have a 15–25% lower IOP compared to their wild-type (WT) counterparts that appears to be the result of enhanced aqueous drainage using an indirect measurement technique [35,36]. *SPARC -/-* mice also use more of the TM for outflow and exhibited decreased collagen fibril diameter in the JCT TM [36]. Although we have previously described the physiologic effects of SPARC deletion on IOP and central corneal thickness [35], the qualitative effect of SPARC deletion on the mouse JCT ECM is not yet known.

In this study we determined how SPARC expression affects outflow facility in mice. We hypothesized that SPARC regulates outflow facility by shifting the balance of ECM synthesis and turnover in the TM. We measured the outflow facility of *SPARC* -/- and WT mice using more direct measurement techniques and assessed the relative expression of selected structural ECM proteins in TM via immunohistochemistry (IHC) and *in vitro* using cultured murine TM endothelial cells.

## Materials and methods

### Animal husbandry and handling

Animal procedures were conducted in compliance with the ARVO Statement for the Use of Animals in Ophthalmic and Vision Research. The protocol was approved by the Case Western Reserve University Institutional Animal Care and Use Committee (Protocol Number: 2013–0166). All procedures were carried out under ketamine/xylazine anesthesia, and all efforts were made to minimize suffering. To measure IOP and assess aqueous humor dynamics, animals were anesthetized with an intraperitoneal (IP) injection (0.1–0.2 ml/20 g body mass) of an anesthetic cocktail containing ketamine (16.5 mg/ml) and xylazine (1.65 mg/ml) which was provided by the animal facility. *SPARC -/-* mice and their wild type (C57BL6-SV129) strain had been previously generated [35]. The mice were bred at the Animal Resource Center of Case Western Reserve University. Equal numbers of male and female mice between 6 and 8 weeks in age were used. All animals were maintained on a 12-hour light/12-hour dark cycle (on 7:00 AM, off 7:00 PM) with food and water available *ad libitum*. Mice were sacrificed by anesthetic overdose followed by cervical neck dislocation once confirmed to be non-responsive in accordance to our approved IACUC protocol.

### Intraocular Pressure (IOP) measurement

The Tonolab (Icare, Vantaa, Finland) was used to measure IOP through rebound tonometry. Our technique has been described in detail [35–37]. Briefly, mice were anesthetized via an I.P. injection of a cocktail of ketamine/xylazine. Following anesthesia, the animal was placed on a movable stand (BrandTech Support Jack; BrandTechScientific, Essex, CT) with its nose inside the facemask. The Tonolab (Colonial Medical Supply, Franconia, NH) was fixed horizontally, and a remote pedal was used to actuate measurements to eliminate potential artifacts caused by manual handling of the device. The mouse was positioned to allow the probe to contact the central cornea perpendicularly. Three sets of six measurements each were made, and the modes of each set were averaged; this value was recorded as the IOP. All measurements were conducted between 11 AM and 3 PM to minimize any potential artifact from circadian variability [16,35]. Additionally, the IOP was measured between 4 and 7 minutes after anesthetic injections on either the left or right eye (randomly chosen for each mouse) without adjusting the stand or tonometer. The IOP was not measured before 4 minutes because mice were not sufficiently anesthetized before this time to allow corneal contact with the probe [34]. Also, prior to 4 minutes, ketamine may have induced a temporary increase in IOP [38].

### Episcleral venous pressure (P_e_) measurement

We estimated P_e_ using an adapted method that was previously described by two separate groups, Weinreb RN *et al*. and Millar JC *et al*. [39,40]. After the mice were anesthetized, a 35-gauge microneedle was inserted into the anterior chamber. The three-way valve was switched so that the manometer reservoir was open to the eye; OD or OS was randomly selected for measurement. The reservoir was then lowered at a rate of 1 mmHg/min until reflux of blood from the episcleral veins into Schlemm’s canal was observed [39]. While monitoring the limbus under a professional ophthalmoscope (Keeler Instruments USA, Inc., Broomhall, PA), we recorded the pressure at which the reflux of blood was first observed and designated it the P_e_. The measurement procedure was repeated twice in each eye.

### Outflow facility (C) measurement

We evaluated outflow facility using a previously published method [40]. The three-way valve was switched to close the reservoir and open the microdialysis infusion pump (SP101i Microdialysis Infusion Pump, World Precision Instruments (WPI), Sarasota, FL, USA) connected to 3 mL syringes. Air bubbles were washed out at a flow rate of 300 μl/min, and the pressure was monitored using LabVIEW software. After adjusting the pressure manometrically to pre-cannulation values (~10 mmHg) and allowing 10–15 minutes for pressure to stabilize, eyes were then initially perfused at a flow rate of 0.1 μL/min and the system was allowed to run for 12 minutes to achieve stable pressure. The flow rate was then increased to 0.2, 0.3, 0.4, and 0.5 μL/min, and the stabilized pressures at each flow rate were recorded. C (μL/min/mmHg) was calculated as the reciprocal of the slope of the respective pressure-flow rate curves. An eye showing a regression value (R^2^) less than 0.9 was excluded from analysis. After measurement of C, the animal was euthanized via anesthetic overdose. After approximately 30 minutes, C was measured again (C_dead_). We assumed that after death (absence of heartbeat), aqueous production and P_e_ would both be reduced to 0.

### Calculating uveoscleral outflow (F_u_) and eliminating aqueous formation rate (F_in_)

We referenced the same modified Goldmann equation as reported by Millar *et al*. [40]. As described, the IOP and P_e_ in each eye were measured directly by rebound tonometry and manometric methods, respectively. When the microneedle was inserted into the anterior chamber and the infusion pump was switched on, the equation is as follows:

$$IOP_{p}=\left[\left(F_{p}+F_{in}‐F_{u}\right)/C\right]+P_{e}$$

where IOP_p_ is IOP when the infusion pump is on, and F_p_ is the flow rate incurred by the infusion pump. By adjusting F_p_ to different values (0.1–0.5 μL/min), C was determined as the reciprocal of the slope of the IOP_p_/F_p_ regression line. After mice were euthanized, F_in_ and P_e_ were assumed to be 0. The euthanized animals were perfused once again to determine C_dead_. F_u_ and F_in_ values for live mice were then estimated for live mice by calculation, as described by Millar, Clark and Pang (2011) [40].

### Comparison of F_u(live)_ and F_u(dead)_

To compare F_u_ in live eyes with F_u_ in eyes immediately after death, F_u_ was measured directly as described previously [40]. Briefly, three live *SPARC -/-* mice, three live WT mice, three dead *SPARC -/-* mice, and three dead WT mice freshly euthanized by anesthetic overdose (i.e. six eyes in each group) were used. For the live mice, a 35-gauge needle was inserted into the anterior chamber for perfusion with fluorescein isothiocyanate (FITC)-dextran (10–4 M; 7000 ng/μL; Sigma Chemical Co, St. Louis, MO) at a flow rate of 0.5 μL/min for 10 minutes. The live mice were then euthanized by anesthetic overdose. For the dead mice, the carcasses were perfused with sterile saline for 30 minutes immediately after death, after which the anterior chambers were perfused in the same manner as the live mice. The eyes were then enucleated and dissected to exclude the cornea and TM. The remaining portions of the eyes were dissected into three parts: the lens, the vitreous, and the retina/choroid/iris-ciliary body/scleral shell. Each part was homogenized and centrifuged in PBS. The supernatant was used to measure the fluorescence intensity (excitation 492 nm, emission 518 nm). F_u_ for each eye was calculated as:

$$F_{u}\left(\mu L/min\right)=[\sum \left(a\times b\right)]/[initial\;concentration\;of\;FITC‐dextran\left(ng/\mu L\times T)\right]$$

where *a* is the volume of the supernatant analyzed (mL), *b* is the FITC-dextran concentration in the sample (ng/mL), and *T* is perfusion time (min).

### Immunohistochemistry

Paraffin-embedded TM tissue slides were deparaffinized in xylene for 15 minutes and incubated with xylene for a second 15 minute period. Sections were subsequently hydrated with ethanol dilutions (100%, 95%, and 70%). The tissue was blocked in 5% bovine serum albumin for 1 hour at room temperature (RT), then permeabilized using 0.2% Triton X-100. The primary antibody was applied at 1:100 dilution overnight at 4°C (Table 1). Slides were subsequently washed with 1×PBS-T, and secondary antibodies were applied at 1:200 for 1 hour at RT (Table 1). After two additional washes, a slide cover was mounted with SlowFade Gold antifade reagent with DAPI (S36938; Life technologies, Eugene, OR). The tissue was imaged using a Leica DMI 6000 B inverted microscope (at 40x) and a Retiga EXi Aqua Blue camera (Q-imaging, Vancouver, British Columbia, Canada). Three rectangular areas of equal size within the TM were selected at random for each section, and Metamorph Imaging Software (Molecular Devices, Downingtown, PA) was used to calculate the average fluorescence through the 488 nm and 594 nm channels.

**Table 1:** Antibodies used for immunoblotting and immunofluorescence.

Primary antibody		Company	Host Species	Dilution	Secondary Antibody	Company	Dilution
**Immunoblot**							
**ECM**	**Collagen I**	**Rockland**	**Rabbit**	**1:1000**	**IRDye 800 anti-rabbit IgG**	**Rockland**	**1:10000**
	**Collagen IV**	**Rockland**	**Rabbit**	**1:1000**	**IRDye 800 anti-rabbit IgG**	**Rockland**	**1:10000**
	**Collagen VI**	**Rockland**	**Rabbit**	**1:1000**	**IRDye 800 anti-rabbit IgG**	**Rockland**	**1:10000**
	**Fibronectin**	**Sigma-Aldrich**	**Rabbit**	**1:1000**	**IRDye 800 anti-rabbit IgG**	**Rockland**	**1:10000**
	**Laminin**	**Sigma-Aldrich**	**Mouse**	**1:1000**	**IRDye 700 anti-mouse IgG**	**Rockland**	**1:10000**
**Matricellular**	**Hevin**	**R&D Systems**	**Goat**	**1:1000**	**IRDye 800 anti-goat IgG**	**Rockland**	**1:10000**
	**Osteopontin**	**R&D Systems**	**Goat**	**1:1000**	**IRDye 800 anti-goat IgG**	**Rockland**	**1:10000**
	**SPARC**	**Haematologic Tech**	**Mouse**	**1:10000**	**IRDye 700 anti-mouse IgG**	**Rockland**	**1:10000**
	**TNC**	**Abcam**	**Rabbit**	**1:1000**	**IRDye 800 anti-rabbit IgG**	**Rockland**	**1:10000**
	**TNX**	**Protein Tech**	**Rabbit**	**1:1000**	**IRDye 800 anti-rabbit IgG**	**Rockland**	**1:10000**
	**TSP-1**	**Invitrogen**	**Mouse**	**1:1000**	**IRDye 700 anti-mouse IgG**	**Rockland**	**1:10000**
	**TSP-2**	**BD biosciences**	**Mouse**	**1:1000**	**IRDye 700 anti-mouse IgG**	**Rockland**	**1:10000**
**Others**	**PAI-1**	**Abcam**	**Rabbit**	**1:1000**	**IRDye 800 anti-rabbit IgG**	**Rockland**	**1:10000**
	**β-Actin**	**R&D Systems**	**Rabbit**	**1:1000**	**IRDye 800 anti-rabbit IgG**	**Rockland**	**1:10000**
**Immunofluorescence**							
**ECMs**	**Collagen I**	**Rockland**	**Rabbit**	**1:100**	**Goat anti-rabbit 594**	**Molecular Probes**	**1:200**
	**Collagen IV**	**EMD Millipore**	**Rabbit**	**1:100**	**Goat anti-rabbit 594**	**Molecular Probes**	**1:200**
	**Collagen VI**	**Sigma Aldrich**	**Rabbit**	**1:100**	**Goat anti-rabbit 594**	**Molecular Probes**	**1:200**
	**Fibronectin**	**Abcam**	**Rabbit**	**1:100**	**Goat anti-rabbit 594**	**Molecular Probes**	**1:200**
** **	**Laminin**	**EMD Millipore**	**Rabbit**	**1:100**	**Goat anti-rabbit 594**	**Molecular Probes**	**1:200**
**Matricellular**	**Hevin**	**Proteintech**	**Rabbit**	**1:100**	**Goat anti-rabbit 594**	**Molecular Probes**	**1:200**
	**Osteopontin**	**R&D Systems**	**Goat**	**1:100**	**Donkey anti-goat 594**	**Molecular Probes**	**1:200**
	**SPARC**	**R&D Systems**	**Rat**	**1:100**	**Goat anti-rat 488**	**Molecular Probes**	**1:200**
	**TNC**	**Abcam**	**Rabbit**	**1:100**	**Goat anti-rabbit 594**	**Molecular Probes**	**1:200**
	**TNX**	**Protein Tech**	**Rabbit**	**1:100**	**Goat anti-rabbit 594**	**Molecular Probes**	**1:200**
	**TSP-1**	**Neomarkers**	**Mouse**	**1:100**	**Goat anti-mouse 594**	**Molecular Probes**	**1:200**
** **	**TSP-2**	**BD biosciences**	**Mouse**	**1:100**	**Goat anti-mouse 594**	**Molecular Probes**	**1:200**
**Others**	**PAI-1**	**Abcam**	**Rabbit**	**1:100**	**Goat anti-rabbit 594**	**Molecular Probes**	**1:200**

#### Preparation of Murine TM (MTM) cells

Murine TM cells were harvested using a published methodology from *SPARC* -/- and WT eyes [41]. Briefly, 3–4 month old mice were anesthetized by intraperitoneal (IP) injection of the ketamine/xylazine mixture. They were subsequently placed on a stereotaxic mouse adaptor, and the head was externally secured with two jaw holder cuffs as well as a tooth bar and nose clamp (Stoelting Co, Wood Dale, IL). A 30-gauge needle attached to a 10 μL syringe was mounted onto a microsyringe pump (World Precision Instruments, Inc., Sarasota FL). The pump was mounted to a micromanipulator (World Precision Instruments, Inc.). Under 35x stereotaxic magnification (Kent Scientific Co., Torrington, CT), proptosis of the eye was produced by mild pressure over the medial and lateral canthi. A paracentesis was performed to drain aqueous humor. The syringe was then advanced so that the needle was nearly parallel to the surface of the iris. Only one eye was injected, following the ARVO Statement for the Use of Animals in Ophthalmic and Vision Research policy. Two microliters of magnetic microbeads (Spherotech, Lake Forest, IL) were injected into the anterior chamber. After completing the injection, the needle was left in the anterior chamber for 5 minutes before removal. To isolate MTM cells, we used a previously published method [41]. Briefly, mice were sacrificed 7 days after injection and the injected eyes were enucleated and dissected to remove the retina, choroid, vitreous, and lens. Tissue was pooled from 12 mice and digested in 4 mg/mL collagenase A (Worthington Biochemical Corporation, Lakewood Township, NJ) with 4 mg/mL BSA dissolved in PBS. It was incubated at 37°C for 2–4 hours and then attached to a magnet. Cells that did not phagocytize the beads were aspirated from the tube and replaced with fresh media. Resuspended cells were passed through a 100 μm cell strainer (Thermo Scientific, Worcester, MA) and then centrifuged at 600g for 10 minutes. The cell pellet was resuspended in 1 mL media and seeded into a 48-well plate with approximately 500 μL of cell suspension per well.

### Immunoblotting

Our immunoblotting technique has previously been described in detail [21]. The conditioned media (CM) from the cultured MTM cells was harvested, and MTM cells were lysed with 1x radioimmunoprecipitation assay (RIPA) buffer. The conditioned media was concentrated 30-fold using a 10 kDa centrifugal filter unit (MilliporeSigma, St. Louis, MO). Equal amounts of total protein in conditioned media or cell lysate (CL) were mixed with 6x reducing (CL) or non-reducing (CM) SDS sample buffer (Boston BioProducts, Ashland, MA). SDS-PAGE was used to resolve proteins in the samples on 10% polyacrylamide gels. Proteins were transferred onto nitrocellulose membranes with a pore size of 0.2 μm (Invitrogen, Carlsbad, CA). Membranes were incubated for one hour in 0.5x blocking buffer (Rockland, Limerick, PA) at RT. Primary antibodies were added to the membranes, which were incubated overnight at 4°C (Table 1). Membranes were washed three times with 1x TBS/T for 10 minutes at RT, then incubated with secondary antibodies in 0.5x blocking buffer (Rockland) for one hour at RT (Table 1). Membranes were washed three more times with 1x TBS/T and imaged using the Odyssey Infrared Imaging System (Li-Cor, Lincoln, NE). Odyssey densitometric software was used to analyze relative band intensities.

### Statistical analysis

All data were analyzed in Microsoft Excel (Microsoft, Redmond, WA,). IOP, C, F_in_, and F_u_ in *SPARC* -/- compared to WT mice were calculated and assessed for statistical significance using the 2-tailed unpaired Student’s t-test. Statistical analyses for IHC data were completed using unpaired Student’s t-tests. *p* < 0.05 was considered statistically significant. Additionally, 95% confidence intervals were calculated for each parameter.

## Results

### Comparison of F_u(live)_ and F_u(dead)_ in *SPARC* -/- and WT mice

**Fig 1:**
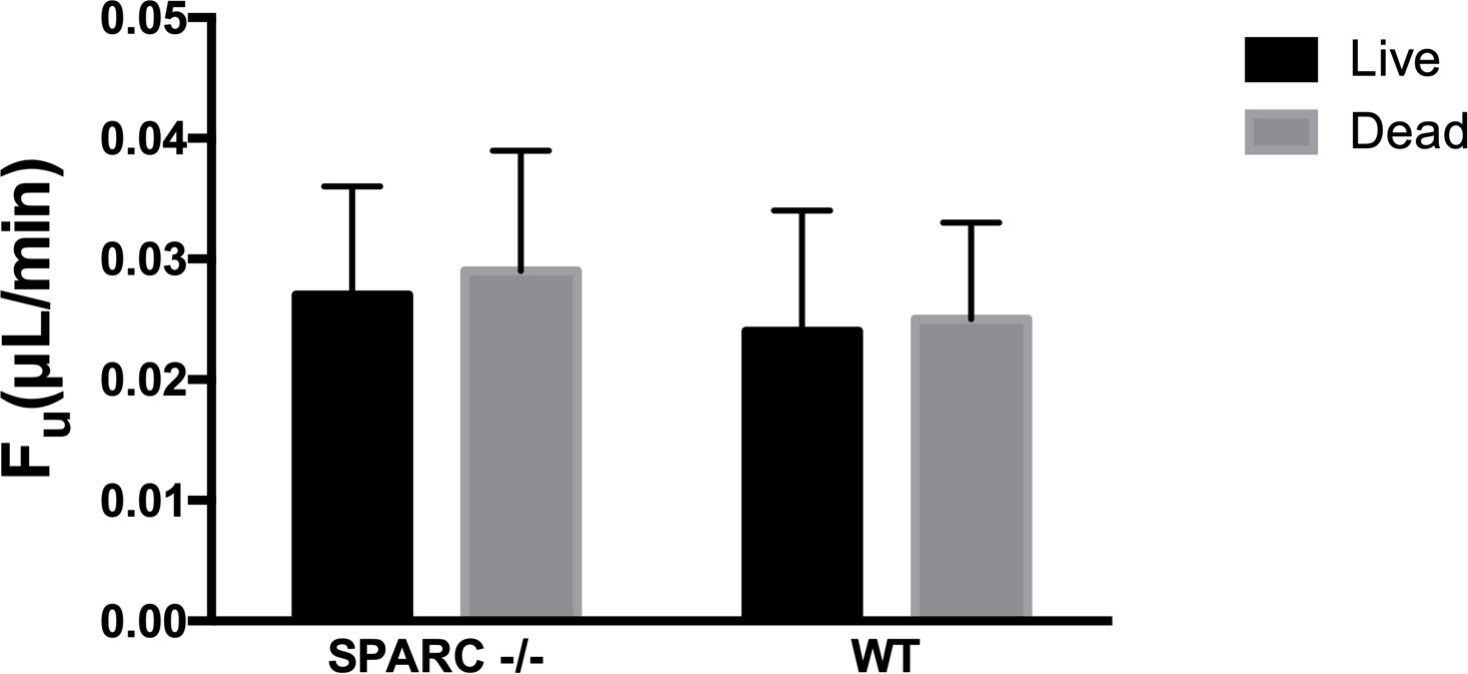
Comparison of uveoscleral outflow in live and dead *SPARC -/-* and WT mice. There were no significant differences between values for F_u_ in live and dead mice (*p* > 0.05).

We used the FITC-dextran perfusion methodology to confirm our assumption that under a constant perfusion rate of 0.5 μL/min, F_u_ is not changed to a significant extent after euthanasia. F_u(live)_ was 0.027 ± 0.009 μL/min (95% confidence interval (CI) 0.018–0.036) for *SPARC* -/- (mean ± SD, n = 6), which was not significantly different from F_u(dead)_ (0.029 ± 0.011 μL/min) (95%CI 0.017–0.041) (*p =* 0.715) (Fig 1). In WT mice, there was no significant difference between F_u(live)_ (0.024 ± 0.010) (95%CI 0.013–0.035) (n = 6) and F_u(dead)_ (0.025 ± 0.008 μL/min) (95%CI 0.017–0.033) (*p =* 0.951).

### Aqueous humor hydrodynamics in *SPARC* -/- and WT mice

**Fig 2:**
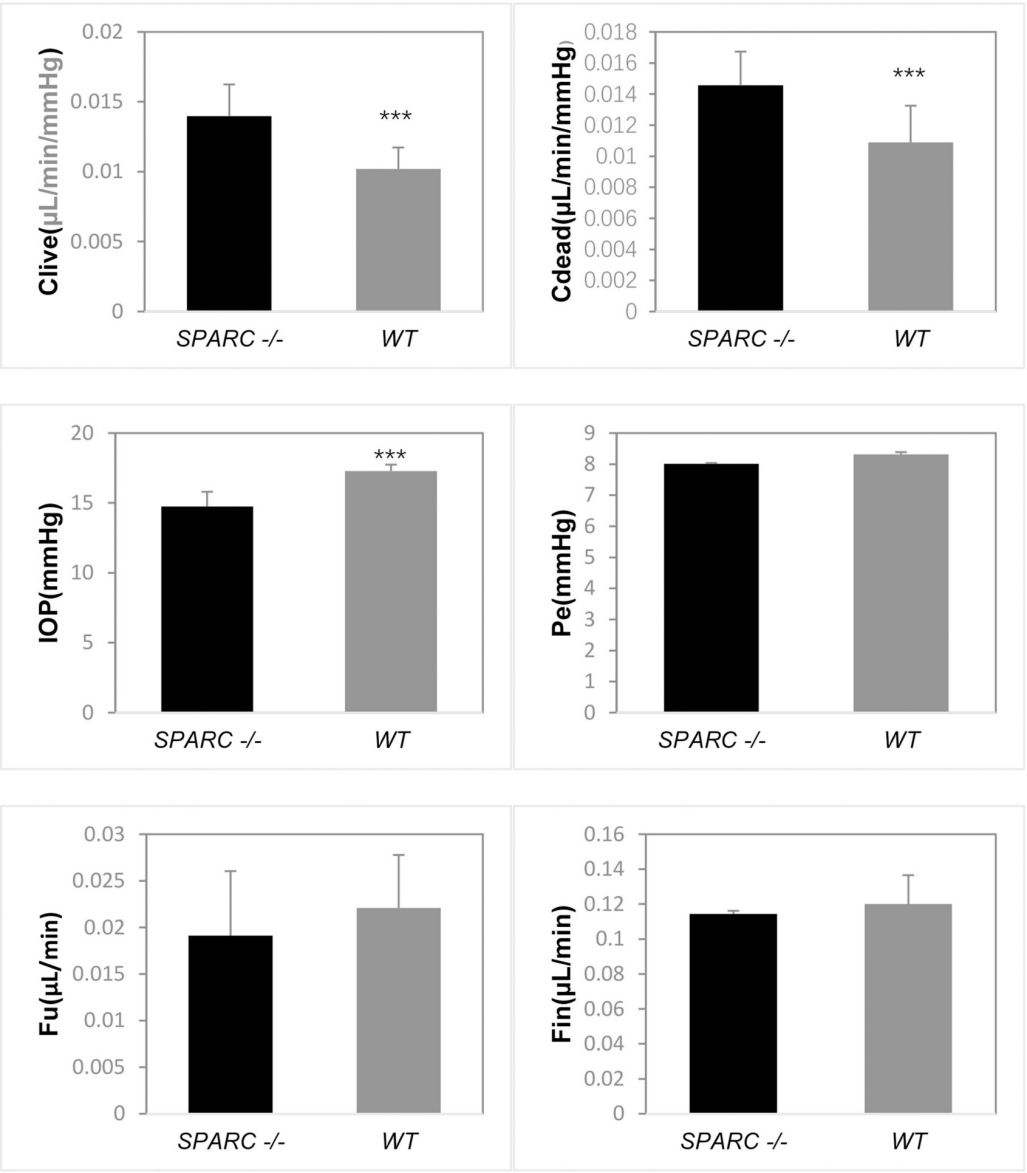
Comparison of parameters of aqueous humor hydrodynamics and IOP in *SPARC* -/- and WT mice. Clive: outflow facility in live mice. Cdead: outflow facility in dead mice. IOP: intraocular pressure. Pe: episcleral venous pressure. Fu: uveoscleral outflow. Fin: aqueous formation rate. WT mice had significantly higher IOP and significantly lower C values. (***, *p* < 0.01).

The IOP, P_e_, and C (before and after euthanasia) of the eight *SPARC* -/- and WT mice eyes were measured, and F_in_ and F_u_ were calculated (Table 2, Fig 2). IOP was 14.7 ± 1.0 mmHg in *SPARC* -/- mice compared to 17.3 ± 0.5 mmHg in WT mice (*p =* 1.98 x 10^−5^, n = 8 in each group). The outflow facility before death (C_live_) in *SPARC* -/- mice (0.014 ± 0.002 μL/min/mmHg), was greater than C_live_ in WT mice (0.010 ± 0.002 μL/min/mmHg) (*p =* 0.002). We compared C_live_ and outflow facility after euthanasia (C_dead_) in *SPARC* -/- and WT mice and found there was no significant difference between C_live_ and C_dead_ in either strain (*p =* 0.376, *p =* 0.548, respectively). P_e_ values were the same in *SPARC* -/- and WT mice, 8.0 ± 0.3 mmHg and 8.3 ± 0.8 mmHg, respectively (*p =* 0.304, n = 8 in each group). F_u_ and F_in_ were the same for both *SPARC* -/- (n = 3) and WT (n = 4) mice (Table 2, Fig 2).

**Table 2:** Parameters of aqueous humor hydrodynamics and IOP in *SPARC* -/- and WT mice.

Parameter	*SPARC* -/-		WT		*p*-value
	Mean ± SD	95% CI	Mean ± SD	95% CI	
C_live_(μL/min/mmHg)	0.014±0.002	0.012–0.016	0.010±0.002	0.009–0.011	0.002 *
C_dead_(μL/min/mmHg)	0.015±0.002	0.013–0.017	0.011±0.002	0.009–0.013	0.006*
IOP(mmHg)	14.7±1.0	13.8–15.6	17.3±0.5	16.9–17.7	1.98E-5*
P_e_(mmHg)	8.0±0.2	7.8–8.2	8.3±0.7	7.7–8.9	0.304
F_u_(μL/min)	0.019±0.007	0.002–0.036	0.022±0.006	0.013–0.031	0.561
F_in_(μL/min)	0.114±0.002	0.110–0.119	0.120±0.016	0.094–0.146	0.591

All values are expressed as mean ± SD, and 95% CI are shown. *p*-values were derived from comparing the parameters in *SPARC* -/- and WT mice using unpaired Student’s t-tests.

* indicates statistical significance.

### Effect of SPARC Deletion on ECM Proteins in *SPARC -/-* and WT mice

**Fig 3:**
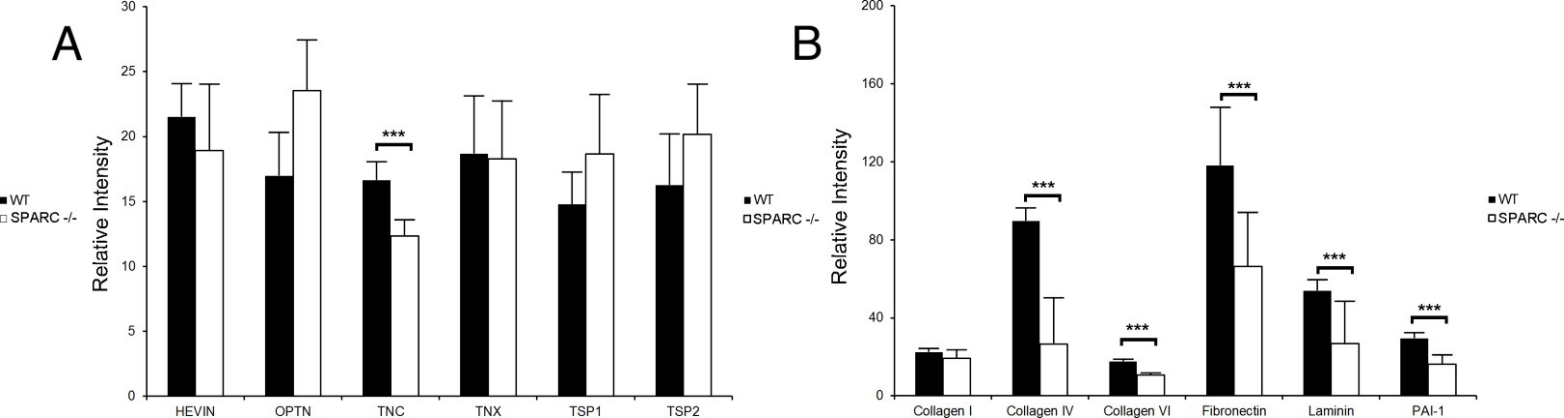
Fluorescence intensities of (A) matricellular and (B) ECM proteins and PAI-1 in WT and *SPARC* -/- mice. WT mice had greater intensities of collagen types IV and VI, fibronectin, laminin, PAI-1, and tenascin-C (***, *p* < 0.05). Error bars represent standard deviations.

**Fig 4:**
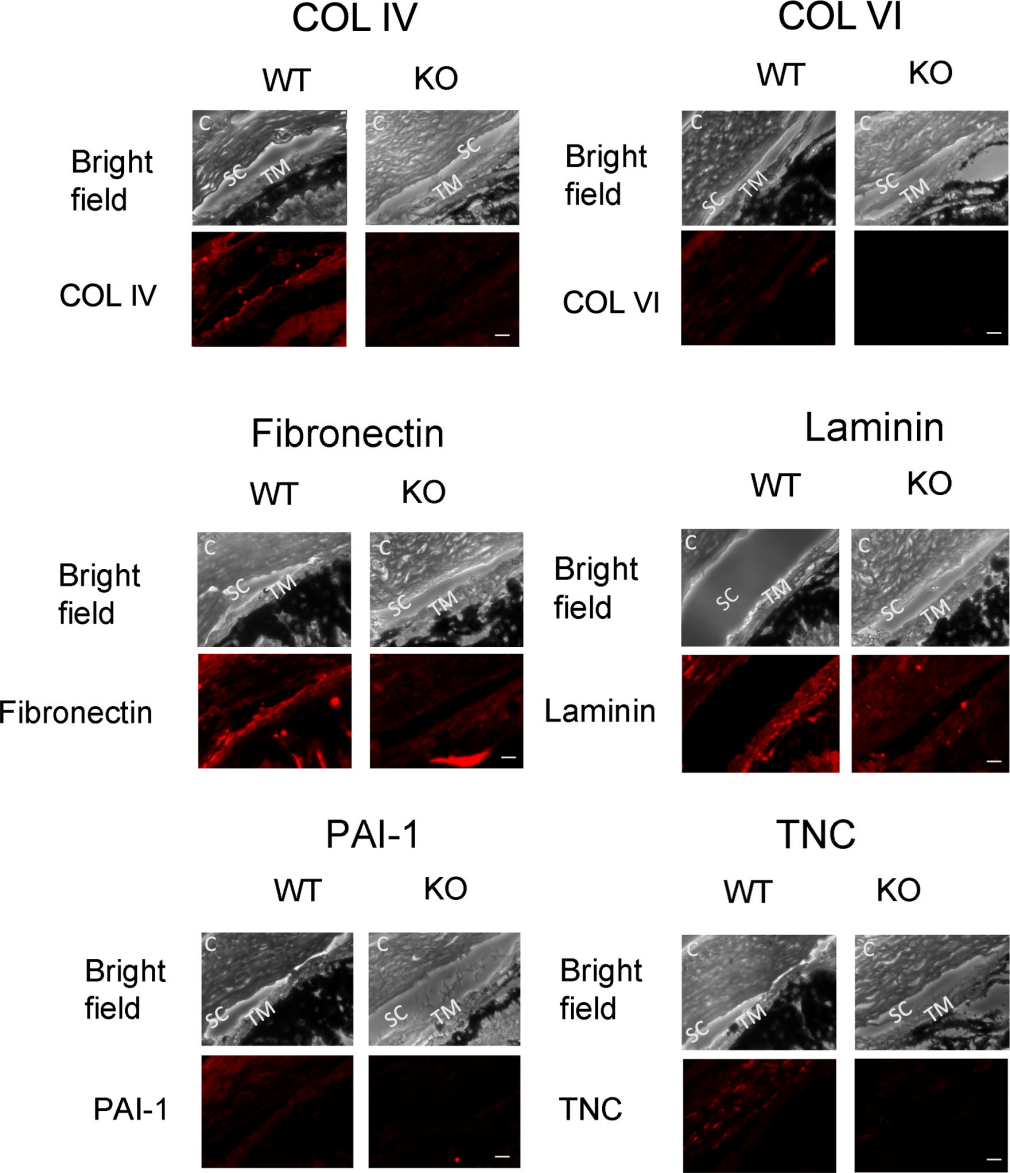
IHC comparison of selected proteins in WT and *SPARC -/-* mice in the JCT. SC: Schlemm’s canal. TM: trabecular meshwork. C: cornea. Collagen types IV and VI, fibronectin, laminin, PAI-1, and TNC were all significantly decreased in *SPARC* -/- mice. Other ECM and matricellular proteins exhibited no significant differences in intensity between WT and *SPARC -/-* mice; these images have been omitted in the interest of space. Not shown: SPARC labeling was negative in all *SPARC -/-* mice. Scale bars: 20 μm.

IHC was performed to investigate changes of ECM proteins in the TM. The fluorescence intensities of collagen IV, collagen VI, fibronectin, laminin, PAI-1, and tenascin-C exhibited significant decreases in *SPARC -/-* mice compared to WT mice (*p* < 0.05 for all). There were no differences in fluorescence intensities between *SPARC* -/- and WT mice for collagen I, hevin, osteopontin, tenascin-X, TSP-1, and TSP-2 (*p* > 0.05). There was no fluorescence staining for SPARC in *SPARC* -/- mice. (Table 3, Figs 3 and 4).

**Table 3:** ECM and matricellular protein percent change in *SPARC -/-* mice compared to WT mice. Percent change in TM tissue was determined through IHC. Percent change in MTM culture was determined through immunoblotting.

**% change in SPARC-KO TM tissue (vs WT)**			
**Target**	**%**	***p*-value**	**n**
**Collagen I**	**-10.08 ± 24.0**	**0.4331**	**4**
**Collagen IV**	**-67.51 ± 14.53**	0.0001*	**4**
**Collagen VI**	**-38.64 ± 7.11**	0.0001*	**4**
**Fibronectin**	**-39.65 ± 33.34**	0.019*	**4**
**Laminin**	**-46.41 ± 21.11**	0.0046*	**4**
**PAI-1**	**-43.29 ± 16.63**	0.002*	**4**
**Hevin**	**-11.29 ± 23.13**	**0.3667**	**4**
**OPTN**	**43.72 ± 39.09**	**0.0666**	**4**
**TNC**	**-25.5 ± 7.85**	0.0006*	**4**
**TNX**	**-19.81 ± 19.78**	**0.092**	**4**
**TSP-1**	**29.52 ± 37.94**	**0.1707**	**4**
**TSP-2**	**27.96 ± 43.99**	**0.2507**	**4**
**% change in SPARC-null MTM culture (vs WT)**			
**Target**	**%**	***p*-value**	**n**
**Collagen I**	**75.67 ± 108.54**	**0.2937**	**3**
**Collagen IV**	**-69.7 ± 22.8**	0.0061*	**3**
**Collagen VI**	**-45.89 ± 13.25**	0.0039*	**3**
**Fibronectin**	**-62.71 ± 6.53**	0.0001*	**5**
**Laminin**	**-5.9 ± 27.21**	**0.6797**	**4**
**PAI-1**	**317.8 ± 50.8**	0.0004*	**3**
**Hevin**	**4.77 ± 13.8**	**0.5816**	**3**
**OPTN**	**110.1 ± 166.8**	**0.235**	**4**
**TNC**	**-76.53 ± 20.26**	0.0028*	**3**
**TNX**	**-37.15 ± 47.4**	**0.2462**	**3**
**TSP-1**	**-13.76 ± 57.23**	**0.554**	**3**
**TSP-2**	**-5.46 ± 59.87**	**0.8821**	**3**

* indicates statistical significance.

The expression of ECM and matricellular proteins from murine TM (MTM) cell culture demonstrated fewer significant changes between WT and *SPARC* -/- mice than the IHC fluorescence. Namely, laminin did not show a statistically significant change in band intensity in western blots of protein from MTM cell cultures (Table 3). Unlike the other proteins investigated, PAI-1 expression changed in opposite directions in the absence of SPARC between *in vivo* and *in vitro* settings; PAI-1 expression was decreased in mice but increased in cell culture. Collagens IV and VI, fibronectin, and tenascin-C were significantly decreased in *SPARC* -/- mice via the MTM method, which is consistent with the findings from the IHC fluorescence method.

## Discussion

We have previously shown that *SPARC -/-* mice have a 15–25% lower IOP and greater areas of TM utilized for filtration compared to wild-type mice [35,36]. Using the technique of constant-flow infusion reported by Millar *et al*. [40], we demonstrated that the differences in IOP between *SPARC -/-* and WT mice are attributable to differences in C values, i.e. outflow facility. In this study, *SPARC -/-* mice showed a 14.7 ± 6.4% decrease in IOP, corresponding to a 37.8% ± 16.1% increase in C value compared to WT mice; the P_e_, F_in_, and F_u_ were unchanged between *SPARC -/-* and WT mice. Regression analysis applied to individual mice revealed a close-to-linear linear correlation between infusion rate and pressure over the range of flow rates studied. As mentioned by Millar *et al*. [40], this technique can also measure C in the eyes of euthanized animals. Combining this value with IOP and P_e_ measurements allows for determination of all aqueous humor hydrodynamics parameters, e.g. F_u_ and F_in_, as described in the Goldmann equation. Millar *et al*. [40] validated their assumptions that euthanasia does not affect C and F_u_. It was assumed that euthanasia reduced F_in_ and P_e_ to zero. Additionally, we used FITC-dextran perfusion to directly measure F_u_ and found that F_u_ was not affected by euthanasia in *SPARC -/-* and WT mice. In our WT mice, aqueous humor production and outflow facility values were similar, but not identical, to values of other mice reported in previous studies [39,40]; differences in mouse strains, measurement techniques, and anesthesia likely account for the differences of values. We and others have found that IOPs and conventional outflow vary among different mouse strains and durations of anesthesia [42–48].

We observed a decrease of collagen types IV and VI, fibronectin, and laminin within the JCT TM of *SPARC -/-* mice relative to WT mice. In MTM cell cultures, these changes were also seen with the exception of laminin. Additionally, PAI-1 was decreased in the JCT TM of *SPARC -/-* mice but increased in MTM cultures from *SPARC -/-* mice. The different results between IHC and MTM cell cultures could be due to a true lack of change in expression, as observed with hevin, TSP-1, and TSP-2, or due to differences in substrate, i.e. cell culture versus *in vivo* setting. Alterations of ECM in JCT have been shown by numerous authors to correlate with IOP changes in various model systems [49–51]. In perfused human cadaveric anterior segments, we found that SPARC overexpression increases IOP and correlates with an increase in collagen IV, fibronectin, and laminin [21]. Thus, it is likely that the observed decreases in collagens and fibronectin are responsible for the reduced outflow resistance. Taken together, the murine data in this study along with our data in perfused cadaveric human anterior segments and TM cell cultures strongly indicate that changes in collagen IV, collagen VI, and fibronectin closely follow changes in SPARC [21].

Our observations of ECM differences in the JCT are consistent with other observations in non-ocular tissues of *SPARC -/-* mice. *SPARC -/-* mice have differences in collagen fibril morphology and less collagen in non-ocular tissues [52]. *SPARC -/-* mice have decreased laminin and collagen IV deposition in renal tissue, decreasing damage from experimental diabetic nephropathy [53]. Similar decreases in interstitial collagen are apparent in hearts and in fat depots of *SPARC* -/- mice [54,55]. We previously reported that collagen fibril diameter was significantly decreased in the JCT of *SPARC -/-* mice, reflecting the importance of SPARC in collagen processing and a potential mechanism by which IOP is reduced in *SPARC* -/- mice [2]. Significant changes in collagen fibril diameter in other tissues of *SPARC* -/- mice have been reported in previous studies [56,57]. For instance, Martinek *et al*. showed that collagen fibrils formed in the absence of SPARC are smaller and more uniform in diameter than those of wild-type animals [58]. Additionally, overexpression of *SPARC* by adenoviral delivery in WT animals subjected to myocardial infarction enhanced collagen assembly in these mice and improved cardiac function [59]. Taken together, these studies provide evidence that SPARC regulates collagen assembly and ECM homeostasis.

It is plausible that other members of the matricellular family may compensate for the loss of SPARC. We found that tenascin-C was decreased in the JCT TM of *SPARC* -/- mice and in MTM cultures from *SPARC -/-* mice. The functional significance of this change is unclear. However, we have previously shown that single gene deletions of TSP-1 and -2 result in a lower IOP in mice [46,60]. Single gene deletions of osteopontin, hevin, and tenascins-C and -X do not alter IOP in mice [37,46,61]. The lack of change of hevin, osteopontin, tenascin-X, TSP-1, and TSP-2 in *SPARC -/-* mice suggests that they may work through SPARC-independent pathways to modulate their effects.

Immunohistological examination revealed decreases in fibronectin and collagen types IV and VI within the TM of *SPARC* -/- mice. Furthermore, immunoblot of MTM cells revealed similar decreases in fibronectin and collagen types IV and VI. The JCT region contains Schlemm's canal (SC) inner wall cells, subendothelial ECM containing an incomplete basement membrane, and TM endothelial cells; these components represent the anatomic location of maximal outflow resistance [62,63]. Regulation of ECM homeostasis has been shown to influence IOP and outflow resistance [9–11,21,64,65]. Collagen IV, fibronectin, and laminin are the main components of the basement membrane of the JCT [66]. An increase of basement membrane components collagen IV and fibronectin within the JCT is a significant structural finding in corticosteroid-induced glaucoma, in which IOP is elevated most likely due to increased TM resistance [67–69]. Decreased permeability is also evident when high glucose and dexamethasone induce collagen IV and laminin in monolayers of TM endothelial cells [70]. This structure-function relationship between ECM and outflow is evident in an inverse manner in our study, where a decrease of ECM components in the JCT seen in *SPARC* -/- mice correlates with increased TM outflow. Therefore, it is likely that ECM components are significant contributors to the mechanism of decreased IOP in *SPARC* -/- mice.

We have demonstrated that the lower IOP of *SPARC* -/- mice is due to greater aqueous humor outflow facility. The observed changes in ECM components in the JCT region provide additional evidence of the importance of ECM homeostasis for IOP regulation. The role of SPARC in influencing these changes in ECM organization and the associated changes in IOP makes SPARC a promising target of further study for its apparent relevance to the pathogenesis of POAG. Future work is planned to characterize the signaling pathways of SPARC in the TM and the mechanisms by which SPARC regulates IOP and JCT ECM homeostasis.

## Supporting information

S1 Dataset Comparison of F_u(live)_ and F_u(dead)_. (XLS)

S2 Dataset Aqueous humor hydrodynamics. (XLS)
